# Direct next-generation sequencing of virus-human mixed samples without pretreatment is favorable to recover virus genome

**DOI:** 10.1186/s13062-016-0105-x

**Published:** 2016-01-12

**Authors:** Dingchen Li, Zongwei Li, Zhe Zhou, Zhen Li, Xinyan Qu, Peisong Xu, Pingkun Zhou, Xiaochen Bo, Ming Ni

**Affiliations:** Department of Biotechnology, Beijing Institute of Radiation Medicine, 27 Taiping Road, Beijing, 100850 People’s Republic of China; Genomics Center of Academy of Military Medical Sciences, 27 Taiping Road, Beijing, 100850 People’s Republic of China; Department of Radiation Toxicology and Oncology, Beijing Key Laboratory for Radiobiology, Beijing Institute of Radiation Medicine, 27 Taiping Road, Beijing, 100850 People’s Republic of China; Department of Research Service, Zhiyuan Inspection Medical Institute, 8 Huazangsi Lane, Hangzhou, 310009 People’s Republic of China

**Keywords:** Pathogen genome recovery, Nonculture, Background Depletion, Whole Transcriptome Amplification, Next-generation sequencing

## Abstract

**Abstract:**

Next-generation sequencing (NGS) enables the recovery of pathogen genomes from clinical samples without the need for culturing. Depletion of host/microbiota components (e.g., ribosomal RNA and poly-A RNA) and whole DNA/cDNA amplification are routine methods to improve recovery results. Using mixtures of human and influenza A virus (H1N1) RNA as a model, we found that background depletion and whole transcriptome amplification introduced biased distributions of read coverage over the H1N1 genome, thereby hampering genome assembly. Influenza serotyping was also affected by pretreatments. We propose that direct sequencing of noncultured samples without pretreatment is a favorable option for pathogen genome recovery applications.

**Reviewer:**

This article was reviewed by Sebastian Maurer-Stroh.

**Electronic supplementary material:**

The online version of this article (doi:10.1186/s13062-016-0105-x) contains supplementary material, which is available to authorized users.

## Findings

Pathogen identification is a critical clinical application [1–3]. Identification methods based on culture have disadvantages, such as long turnaround time, increased biohazard risks, and culture bias. The high-throughput feature of NGS enables the recovery of pathogen genomes from noncultured samples, and offers the potential for highly accurate pathogen identification and rapid clinical diagnoses [4–12]. Many researchers have reported the NGS-based identification of pathogens from various noncultured samples [13–21], such as Old World arenavirus (brain et al.) [17], influenza virus (nasopharyngeal aspirate) [18], norovirus (feces) [18], dengue virus [19], yellow fever virus (serum) [20], Shiga-toxigenic *Escherichia coli* O104:H4 (feces) [21], and most recently, Ebola virus (serum et al.) [13–16].

Two major challenges must be overcome when we seek to recover pathogen genomes from noncultured samples: noise from host and/or microbiota cells, and limited availability of DNA/RNA. Consequently, two pretreatments are usually employed before sequencing noncultured samples: background depletion (BD) to increase the signal-to-noise ratio [22, 23], and alleged unbiased amplification to increase the amount of available nucleic acid in order to meet the requirement of NGS library preparation [24, 25]. Despite of the benefits, how these pretreatments influence pathogen genome recovery during the sequencing of pathogenic DNA/RNA from noncultured samples has not been fully investigated.

### Effects of pretreatments on influenza virus identification

We applied different pretreatments (BD with or without Whole Transcriptome Amplification, abbreviated as WTA) to mixtures of human RNA and influenza A (H1N1) virus RNA, as a noncultured model system, and applied NGS to evaluate the effects of pretreatments on influenza genome recovery (Additional file 1: Figure S1). The four sample pretreatments were as follows: (1) BD, (2) WTA, (3) BD + WTA, and (4) no pretreatment. Effects of amplification time (2 or 8 h) and viral ratio (0.55 or 1.5 % viral RNA within RNA mixtures) were examined. NGS libraries were constructed of samples with different pretreatments. We obtained 12 gigabases of sequence data. After quality control and removal of human reads, the remanent reads were aligned to a dataset consisting of 246,715 flu genome sequences (Additional file 2) for influenza read identification and serotyping.

**Fig. 1:**
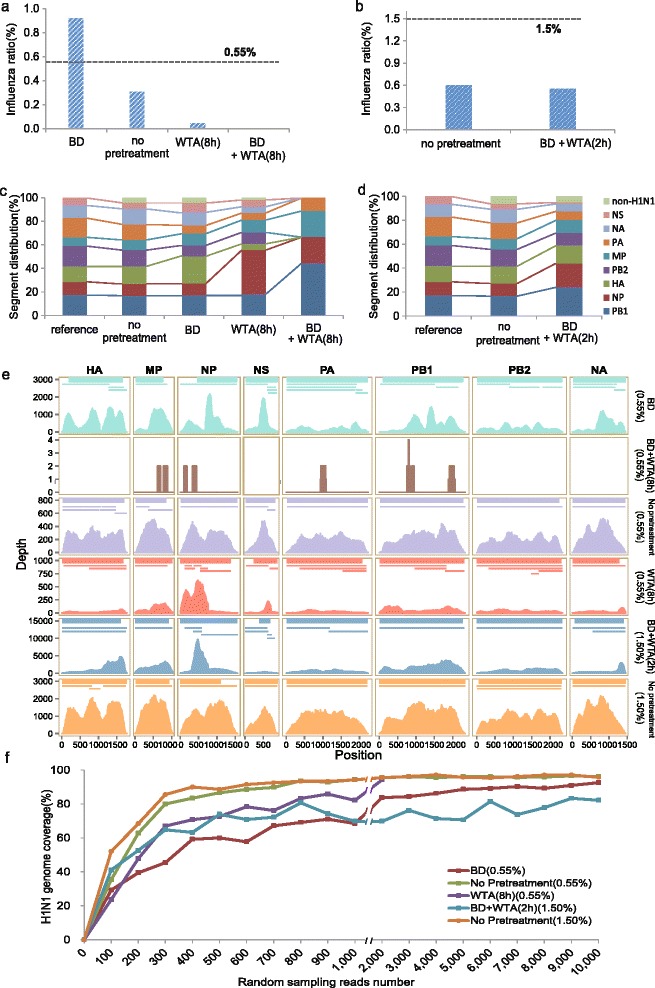
Identification, serotyping and genome recovery of influenza A virus based on reads. Expected proportions of H1N1 within mixed RNA samples were 0.55 % (**a**, and **c**) and 1.5 % (**b**, and **d**). **a** and **b** Percentages of reads aligned to influenza A virus among all reads passing quality control. Black dashed lines denote expected proportion of influenza A virus. **c** and **d** Influenza A virus-aligned read distribution for influenza A viral RNA segments. Influenza A virus-aligned reads of “BD + WTA (8 h)” are insufficient; thus, their distribution is not shown. **e** Site depths based on NGS read alignments on eight H1N1 RNA segments are shown as a filled area graph, colored by experimental condition. Segment names and their positions are labeled at top and bottom, respectively. Assembled contig alignments are denoted by thick lines (Velvet assembly) or thin lines (Trinity assembly) up the site depth profiles. **f** H1N1 genome recovery coverage with increasing numbers of random sampling reads. For a given read number, influenza A virus-aligned reads were randomly sampled 10 times for de novo assembly, and the average coverage values are shown

The influenza ratio, defined as the ratio of the number of influenza A-aligned reads to the total number of reads, ranged from 0 to 0.92 % and was greatly affected by pretreatment (Fig. 1a and b). Ratios from samples without pretreatment were lower than expected proportions (0.31 *vs.* 0.55 %; 0.57 *vs.* 1.5 %), indicating that the NGS library preparation could decrease the viral ratio. The influenza ratio with BD pretreatment was higher than expected (0.92 *vs.* 0.55 %) and approximately 3-fold higher than the ratio without pretreatment. The majority of rest reads were from incomplete removal of host RNA (Additional file 3: Figure S2). It should be addressed that these samples were contaminated with mycoplasma, which accounted for 0.14 to 5.05 % of the total reads (Additional file 2, Additional file 4: Table S1). Although BD could be helpful in viral detection, this treatment decreased the amount of sample RNA from 19.2 to 1.95 ng/μL. As clinical samples (as swab and serum) usually have much lower RNA/DNA content than our model samples, BD alone was an impractical treatment due to the NGS library nucleic acid input requirement.

WTA for 8 h, with or without BD, remarkably decreased the influenza ratio (0.05 % or almost 0). For samples with an expected viral proportion of 1.5 %, we observed comparable influenza ratios of about 0.57 % for the no-pretreatment and BD + 2-h WTA pretreatment. As BD increased the influenza ratio while WTA decreased it, we hypothesized that there was a trade-off for viral detection between BD and WTA, and that the effects were in equilibrium when WTA was 2 h.

Next, we examined the effects of different pretreatments on influenza A viral serotyping. Most influenza reads with these pretreatments were aligned to segments from the H1N1 serotype (Fig. 1c  and d). Reads aligned to other serotypes could be explained by interstrain sequence homology. However, read distributions on eight RNA segments were also biased by the four treatments (Fig. 1c and d). Although BD could increase influenza ratios, this benefit came at the cost of biased distributions compared to the distribution of sample without pretreatment. WTA further exaggerated the bias among different segments. When we focused on HA/NA segments, except for the BD + 8-h WTA pretreatment which produced almost no influenza reads, pretreatments consistently produced remarkable enrichments of H1N1-aligned reads (Additional file 5: Figure S3). This enrichment was observed even for the 8-h WTA pretreatment (without BD), despite that this pretreatment remarkably reduced influenza ratios and caused biased segment distribution.

### Genome recovery efficiency

With an optimized bioinformatics pipeline, influenza-aligned reads were *de novo* assembled, and assembly contigs were re-aligned to the whole flu genome sequences. The reference genome of H1N1 strain A/Changchun/01/2009(H1N1) was aligned with the highest sequence similarity, with eight single nucleotide variations identified and validated by Sanger sequencing (Additional file 6: Table S2, Additional file 7: Table S3). The whole H1N1 genomes were nearly recovered for all pretreatments except BD + 8-h WTA as the best alignments were all assigned to H1N1. Thus, at both the NGS read and assembly levels, pretreatments did not affect accurate serotyping under conditions that produced sufficient influenza reads.

To further explore the effects of different pretreatments on genome recovery efficiency, we compared the corresponding H1N1 genome site sequencing depth profiles (Fig. 1e and Table 1). The read-aligned regions ranged from 78.7 to 98.7 % of H1N1 genome (except BD + 8-h WTA), and sample with no pretreatment produced the best coverage. Furthermore, depth profiles for the no-pretreatment exhibited the best inter and intra- segment evenness compared to these for other pretreatments (Fig. 1e and Additional file 8: Table S4). Notably, BD by host rRNA and poly(A)-tailed transcript removal also induced biased site depth profiles on the H1N1 genome. This alteration might be attributed to the nonspecific hybridization of magnetic bead probes to influenza. WTA pretreatments induced different patterns in depth profiles compared to BD; part of NP segment seemed to be advantageous during amplification. Moreover, the depth profile of BD + 2-h WTA indicated that the pattern of read alignment bias was dominated by WTA. The analysis of coefficient of variation (CV) on genome sequencing depth indicated that NP, NA and NS were three segments with higher biased coverage (Additional file 8: Table S4). Besides, we explored the possible nucleotide motifs of the missed and over-amplified regions, which are summarized in Additional file 9.

**Table 1:** Genome de novo assembly

Treatment^a^	Trinity assembler			Velvet assembler			IDBA-UD assembler		
	Contig total size (bp)^b^	Genome coverage (%)	H1 + N1 coverage (%)	Contig total size (bp)^b^	Genome coverage (%)	H1 + N1 coverage (%)	Contig total size (bp)^b^	Genome coverage (%)	H1 + N1 coverage (%)
BD (0.55 %)^a^	12,987	95.3	98.3	12,163	89.2	89.5	13,035	95.6	95.0
No pretreatment (0.55 %)^a^	13,351	97.9	98.5	12,993	95.3	96.5	13,077	95.9	95.5
BD + 8-h WTA (0.55 %)^a^	—	—	—	—	—	—	—	—	—
8-h WTA (0.55 %)^a^	12,912	94.7	97.3	12,649	92.8	90.9	10,723	78.7	83.8
BD + 2-h WTA (1.50 %)^a^	13,431	98.5	97.3	12,555	92.1	91.3	13,083	95.9	91.7
No pretreatment (1.50 %)^a^	13,461	98.7	98.5	13,064	95.8	99.6	13,240	97.1	94.4

^a^Expected proportions of H1N1 within mixed RNA samples are indicated in parentheses

^b^Contigs generated by assemblers were aligned to the H1N1 reference genome. Contig total sizes were obtained from aligned contigs. Overlapping contig regions were counted only once

Next, we gradiently and randomly resampled the influenza-aligned reads, and examined the variations of assembly sizes with read number (Fig. 1f). As the read number increased, the samples without pretreatments showed more rapid growth of H1N1 genome coverage than samples with BD and/or WTA pretreatments. About 400 reads could produce an 80 % recovery. About 2000 reads were required for BD treatment. Thus, although BD allowed a higher influenza-aligned read ratio, this benefit was offset by decreased assembly efficiency. Pretreatment with WTA (with or without BD) also obviously reduced the H1N1 genome recovery rate.

## Conclusion

Taken together, direct sequencing of extracted RNA (no pretreatment) provided the best efficacy in recovering H1N1 genomes. Under clinical conditions, the amount of recovered RNA after host removal (without amplification) could be insufficient for NGS library preparation. Moreover, host BD induced bias of NGS read alignment over the viral genome, and thus affected the assembly. On the other hand, WTA increased the total available cDNA but reduced the viral ratio, resulting in reduced sensitivity to detect viral reads, especially for overamplification (8-h WTA) which significantly depleted the viral fraction. Direct sequencing method does not require extra preprocessing steps compared to BD, WTA and many other methods available [22–33], which means fewer experimental procedures, decreased cost, lower technical error rates, and decreased turnaround time. Thus, we propose that direct sequencing without pretreatment is sometimes the optimal solution. These findings will provide input for further studies and clinical implementation.

## Methods

All experiments were approved by the Animal Ethics Committee of the Beijing Institute of Radiation Medicine, in accordance with the regulations of Beijing Administration Office of Laboratory Animals and no patient was involved in the study. Total human RNA was extracted from alveolar adenocarcinoma A549 cells with Invitrogen Trizol Reagent (Life Technologies) and quantified by Qubit 2.0 (Life Technologies). Influenza A virus [34] (A/Changchun/01/2009(H1N1), 13,632 bp) RNA was isolated with the QIAamp Viral RNA Mini Kit (Qiagen) and quantified by quantitative real time PCR (qRT-PCR) with the ABI 7500 PCR system (Applied Biosystems, Inc.) after reverse transcription. Host RNA background depletion (BD) was performed by using an rRNA-hybridization magnetic bead method with the RiboMinus Eukaryote Kit for RNA-Seq (Ribominus Concentration Module, Life Technologies), and further using magnetic beads conjugated to oligo(dT) primers (Illumina) to remove poly(A) tailed transcripts. WTA was performed by using QuantiTect Whole Transcriptome Kit (Qiagen). For samples not requiring amplification, the first and second strand cDNA were generated by using High-Capacity cDNA Reverse Transcription Kits (Applied Biosystems) and the NEBNext mRNA Second-Strand Synthesis Module (New England Biosystems). After purification by the Zymo Purification Kit (Zymo Research), double-stranded DNA (dsDNA) was quantified by Qubit 2.0. DNA inputs of 1 ng were used for multiplex NGS library generation with the Nextera XT DNA Sample Preparation Kit (Illumina). NGS was performed with an Illumina MiSeq platform to generate 150 or 250-bp pair-end reads. All high-quality sequence reads data have been submitted to the NCBI Sequence Read Archive (accession number SRP059219). Raw NGS reads were filtered with quality cutoffs of at least 50 % read bases with quality of Q20 or better, fewer than 10 % N bases, and fewer than 14 continuous N bases. Reads were firstly mapped to the human genome (hg19) and the unaligned reads were then aligned to a dataset including reference genomes of Mycoplasma (313 sequences, NCBI genome database), bacterial (3022 sequences, NCBI genome database), flu (246,715 sequences, EpiFlu, http://platform.gisaid.org and NCBI Nucleotide database, Additional file 2), other viral (1,757,357 sequences, NCBI genome database), and the whole NCBI nucleotide (nt) database by using Bowtie2 [35] (v2.1.0) in the end-to-end, paired-end mode and BLASTn [36]. Metagenomics analysis was carried out by using PathSeq [37] pipeline and Kraken [38]. *De novo* assembly was carried out by using Trinity[39], IDBA-UD [40] and Velvet (v1.2.10) [41]. Particularly for Velvet and IDBA-UD assembling, k-mer lengths were scanned from 9 to 123, and optimal lengths with the largest N50 length were selected. Assembly contigs were aligned to reference segments by using Blastn with a required *E*-value of less than 10^−5^. With the median site sequencing depth (denoted as *D*) for a sample as a baseline, the region with sequencing depth between 50–150 % *D*, < 50 % *D* and > 150 % *D* were defined as uniform, missed and over-amplified regions, respectively. Nucleotide motif discovery was performed by using MEME Suite 4.10.2 [42] and FIMO (*E*-value < 10^−4^) [43] on missed and over-amplified regions for each sample with pretreatment. Influenza-aligned reads were randomly sampled at a step size of 100 or 1000 and then assembled by Velvet; the sampling was repeated 10 times.

## Reviewer’ comments

### Reviewer’s report: Sebastian Maurer-Stroh (Bioinformatics Institute, A*STAR, Singapore)

The advent of next generation sequencing methods clearly has increased the pace with which we can get genome sequences from all possible sources. While the fast moving technological aspects receive broad attention, the accompanying methods for sample preparation, pretreatment and library generation are often neglected although in many situations these can be pivotal for the outcome. This paper offers a welcome different focus on exactly these factors. While the synthetic mixture of human cellular and viral RNA has advantages for quantitative comparisons of the pretreatment methods, one still needs to caution that such sample mixture will have distinct properties from an actual clinical sample (e.g. swab) with all its other material and possible additional biases. The comparison and result is quite clear and the take home message is that there is a big influence coming from the pretreatment which many would have suspected but very few studied and quantified. I would not necessarily say that these results mean one is always better off sequencing clinical samples directly but rather one should carefully consider and study effects of sample pretreatments.

- The big question to me is: if the ratio of influenza reads even after background RNA depletion is so small (<2 %), where are all the other reads from? Incompletely removed host RNA or Bacteria and their phages? Sending these reads through a metagenomics pipeline (e.g. Kraken) may be an interesting idea to follow this up, possibly in future (a word of caution: viral metagenomics remains a challenging task, by own experience, different methods can find different viruses in supposedly single virus samples).

Author’s response: Many thanks for this constructive comment. We accordingly have analyzed the components of total reads by aligning them to human reference genome (hg19), bacterial reference genomes and viral reference genomes, and the results and detailed methods are shown in Additional file 3: Figure S2. Although the influenza ratio increased after background RNA depletion, the majority of rest reads were still from incomplete removed host RNA. As the viral fraction of interest was very small in total RNA, the incompletely depleted host RNA would still be dominant in samples after the pretreatment of background depletion. For instance, the host rRNA ratios were 10.37 and 12.48 % for the samples with BD (0.55 %) and BD + 8-h WTA (0.55 %) pretreatments, respectively. This is consistent with the results of other studies, in which the host rRNA reads ratio account for about 10–40 % after host rRNA removal [23, 26]. Besides, we have carried out metagenomics analysis by using PathSeq [38] pipeline. However, we have not found any confident evidence of bacterial existence, which is understandable as we used cell-line and cultured viruses as study objects. We indeed detected some endogenous retroviruses, which should be inserted in human genome. To sum up, we conclude that the majority of rest reads were from incomplete removal of host RNA.

- From the viro-biological point of view, A549 cells, although commonly used to study influenza virus host interactions, are not the best cells to get high viral titres for example compared to MDCK cells but this is not a problem for this study where a challenging setup is anyways appreciated.

Author’s response: We agree with reviewer’s comment. High viral titres is much favorable for viral identification or viral genome recovery by using NGS technology. Nonetheless, a challenging setup might be more like clinical samples (i.e., swabs and serum) which could have very low viral titres. In this study, we believe that the selection of cell-line would not affect the qualitative result.

- From the Bioinformatics software view, Trinity and Velvet for assembly may not be ideal depending on the k-mer length relative to the gap size. I would also try IDBA-UD which simultaneously uses long and short k-mer lengths but in this case there may not be much difference in the conclusions.

Author’s response: Thanks for the comment. We have employed IDBA-UD to re-assemble the H1N1-aligned reads. However, as the reviewer mentioned, we did not observe much difference in assemblies produced by IDBA-UD compared with those by Trinity and Velvet (Table 1). In the case of this study, Trinity still had the best performance among the assemblers. We have updated Table 1 and corresponding manuscript, which included the results by IDBA-UD.

- Another follow-up or extension of this work would be to statistically analyse both missed and overamplified nucleotide motifs with the different approaches to potentially get ideas how to unbias pretreatment methods better in future.

Author’s response: Thanks for the comment. First, we employed the concept of uniformity to determine missed and regions over-amplified. In detail, with the median site sequencing depth (denoted as *D*) for a sample as a baseline, we selected the region with sequencing depth between 50–150 % *D* as uniform region, whose ratio in genome was the uniformity. The missed and over-amplified regions were defined with site depth < 50 % *D* and > 150 % *D*, respectively. It should be addressed that we also examined uniformity with other thresholds (i.e., 40–160 % *D* or 80–120 % *D*), and the samples without pretreatment consistently had the highest uniformity compared these with BD and/or WTA (data not shown). Then, by using the MEME Suite 4.10.2 [39], we performed calculation of nucleotide motif discovery respectively on missed and over-amplified regions for each sample with pretreatment. The discovered motifs were re-aligned to the H1N1 genome by FIMO [40] (*E*-value < 10^−4^), and their occurrences on the whole genome and missed/over-amplified regions were both obtained. Finally, we selected 10 motifs significantly enriched in missed or over-amplified region (Fisher’s exact test, *p* < 0.05) in the three samples with BD and/or WTA pretreatment, which are shown in Additional file 9. We hope this result could be a hint to improve pretreatment methods in the future.

Furthermore, to quantitatively evaluate which H1N1 segments were more unbiased sequenced, we calculated coefficient of variation (CV) [26, 28] of site sequencing depth for whole H1N1 genome and each segment (Additional file 8: Table S4). The samples with no-pretreatment have remarkable smaller CVs (~0.45) on genome compared with these with pretreatments (1.00–1.59), and no-pretreatment also derived significantly smaller CVs of segments (Additional file 8: Table S4, Wilcoxon rank sum test, *p* < 0.05). Among these segments, we found NA, NP and NS were more likely to have biased sequencing coverage by pretreatments (CV > 1), and they might be paid more attention in future pretreatment.

- Many thanks for responding to my comments in detail and adding several further analyses that were needed to interpret the results better. However, with more results available it is now clear that there is a big problem which may be challenging to be resolved. While checking some of the results for the missed motifs after background depletion in new Additional file 6: Table S2 I noticed that the identified sequence motifs appear to match to A/California/07/2009(H1N1) [the H1N1 from the 2009 swine flu pandemic] rather than A/FM/1/47(H1N1) [an old reference H1N1 strain from 1947] which was mentioned to have been used in the method section. As you should know, there are several very different H1N1 strains known. Adding to the confusion, the associated SRA accession at NCBI is annotated taxonomically suggesting the virus is a mouse-adapted version “Influenza A virus (A/Fort Monmouth/1/1947-mouse adapted(H1N1))” for which no complete reference genome exists in the databases (only some segments). To get a clearer picture, I downloaded and reanalyzed your raw data (assembly and metagenomics for the 1.5 % no treatment SRR2054788 and 1.5 % double treated SRR2054787 sample, respectively). The influenza virus in your samples is in fact a recent H1N1 pdm09 virus (it is most similar to A/Changchun/01/2009(H1N1)), so your method description and the taxonomy annotation submitted to NCBI is wrong. Consequently, the coverage results (Table 1, Fig. 1e) etc require to use a matching genome to be accurate (and all database accessions of used references need to be properly listed). Furthermore, metagenomics analysis suggests a clear contamination with Mycoplasma for both reanalyzed samples which makes up the majority of non-host reads (metagenomics was checked with consensus from gottcha, mini-kraken, metaphlan and bwa readmapping to make sure it is not a spurious result, curious that your analysis with PathSeq did not pick this up). It may have to be established on clean cells that the effects with and without treatment are not influenced by the dominance of Mycoplasma reads or fully characterize its presence and include and discuss it as additional factor inherent to the existing data and analysis. Obviously, with the wrong strains mentioned, potentially wrong references used for analysis and serious undeclared cell contamination this work is not up to any scientific standards for publication. Nevertheless, the basic idea of the work is still good and the principal conclusions may not be much affected after all but it is of critical importance to provide accurate descriptions of the experiments to ensure correctness and reproducability of the results.

Author’s response: Thank you for your reviewing our manuscript again. We are very grateful that you pointed out the mistakes we failed to notice. According to your comments, we have checked and confirmed the virus strain (A/Changchun/01/2009(H1N1)) by using Sanger sequencing. We have re-performed all calculations in this study with updated reference datasets, and updated the corresponding results. Your question on mycoplasma contamination is important. We have actually found it in our samples through PathSeq analysis, but did not pay enough attention and categorized it as component of “others”. We apologize for this inappropriate opinion, and have carefully analyze the presence of the mycoplasma. We have added descriptions in manuscript and additional files to fully characterize the presence of mycoplasma. The results based on the new calculations and analyses show that the principal conclusions of this study remain unaffected. Please see the detailed report and also review the revised manuscript.

We made a mistake about the information of H1N1 strains used in this study, and we are very grateful that the reviewer pointed it out. The strain has been confirmed to be A/Changchun/01/2009(H1N1) rather than A/FM/1/47(H1N1). We have designed PCR primers (Additional file 6: Table S2) and sequenced the full genome of the strain we used by Sanger sequencing. The sequences obtained were consistent with the assembly based on NGS results, and we aligned them to the reference genome of strain A/Changchun/01/2009(H1N1) (accession No. JN032403—JN032410, NCBI Nucleotide database) and identified eight single nucleotide variations (Additional file 7: Table S3).

We have corrected the taxonomy annotation of sequencing data submitted to the NCBI SRA, and re-performed the whole computation of this study. In details, as the influenza reference dataset downloaded from EpiFlu does not contain the strain A/Changchun/01/2009(H1N1), we first updated the reference dataset with 118,955 more sequences from NCBI Nucleotide database (Additional file 2). Then, we removed human-aligned reads, and aligned the rest NGS reads to the new dataset of references. Based on influenza-aligned reads we re-performed serotype and statistical analyses as well as *de novo* assembling, and we found that the results were nearly unchanged, and conclusions were consistent with those in previous version of manuscript. The assemblies were also aligned to the new reference dataset, and we found that the reference genome of highest similarity was from the strain A/Changchun/01/2009(H1N1) (The reference we used in previous version of manuscript is A/New York/NHRC0003/2009(H1N1), which has 34 single nucleotide mismatches with reference of strain A/Changchun/01/2009(H1N1)). Finally, with the reference genome of A/Changchun/01/2009(H1N1), we evaluated the assembly statistics such as coverage and sequencing evenness again, and the results also remained nearly the same. Moreover, in theory, with enough sequencing depth and sufficient reference datasets which contained highly homologous sequences from other strains, bioinformatics analyses and results would not depend on the reference genome. Therefore, we suggest that the wrong strain information and reference genome might not affect the conclusion of this study.

We present here the investigation of how we mistook the strain information. Our laboratory had both of the strains while we performed the experiment. We received RNA sample extracted from strain A/Changchun/01/2009(H1N1) from our colleagues, but we were informed of the wrong strain name, A/FM/1/47(H1N1). Unfortunately, the flu reference genome dataset we used (EpiFlu, Additional file 2) did not include genome sequences of A/Changchun/01/2009(H1N1) (which is available in NCBI Nucleotide Database). The most similar strain when we aligned the assembly to reference dataset was A/New York/NHRC0003/2009(H1N1) (genome similarity: 99.5 to 99.9 % for each segment), and we used it as a reference to evaluate viral genome recovery. While we focused on the efficiency of genome recovery, we did not notice that the used reference genome was not from the alleged A/FM/1/47(H1N1). We apologize for the fault we have made, and thank the reviewer again for pointing it out.

We have re-analyzed the missed and over-amplified nucleotide motifs based on the correct reference genome of A/Changchun/01/2009(H1N1). Compared with the previous result (based on strain A/New York/NHRC0003/2009(H1N1)), the identified motifs exhibited some differences, while three motifs presented in new Additional file 9: Table S5 were the same as the previous. The relevant description in text and Additional file 9 have been revised.

We agree to the existence of mycoplasma contamination. Actually, we have observed the content of mycoplasma in the analysis by using PathSeq, but we assigned the mycoplasma to the “others” category (Additional file 3: Figure S2, previous version of revised manuscript). At that time, we thought that mycoplasma was commonly found in cultured cell lines and might need not to be specially addressed, as the main focus of this study are viral pathogens. We admit that it was an inappropriate opinion, and we should fully characterize the presence of mycoplasma as the reviewer suggested. We have revised Additional file 3: Figure S2 to exhibit the detailed distributions of species based NGS read alignments in this study. Especially, ratios of mycoplasma-aligned reads are shown in new Additional file 4. The rations were obtained by both aligning NGS reads (after removal of the host-aligned reads) to a dataset composed of 313 mycoplasma genome sequences and metagenomics analyses (Additional file 2). Among these samples, mycoplasma-aligned reads in total reads account from 0.14 to 1.8 % (average 0.97 %), except the sample of BD + 2 h-WTA (1.5 %) whose mycoplasma ratio achieved 5.05 %. We speculate that the high Mycoplasma-aligned ratio could be mainly ascribed to the pre-treatments.

A recent paper by Anthony O. Olarerin-George and John B. Hogenesch reported a large scale analysis of RNA-seq data from 9395 rodent and primate samples from 884 series, and found 11 % of the series with cultured samples were contaminated by mycoplasma (Assessing the prevalence of mycoplasma contamination in cell culture via a survey of NCBI’s RNA-seq archive, Nucleic Acids Research, 2015, 43, 2535). The contamination ratios are ranged from 0.01 to 14.43 % (mean = 1.44 %, median = 2.15 %), while the top 20 series with the highest mycoplasma reads ratio include top peer-reviewed journals such as Nature, Cell, PNAS, Genome Research, RNA and Nucleic Acids Research. Another important result of their investigation is an identification of 61 host genes significantly associated with mycoplasma-mapped read counts. In our study, we build model samples by mixing RNA from human cell lines and H1N1 strain, and focus on viral genome recovery. Therefore, instead of gene expression, we care more about valid extraction of viral RNA and the reads count occupied by other microorganism such as mycoplasma. According to the result, we obtained valid H1N1-aligned reads in five samples, and the variations in H1N1 ratio could be mostly attributed to different pre-treatments rather than mycoplasma contamination. On the other hand, compared with our mixture model, clinical specimens such as serum and oral swabs would be more complex due to much greater heterogeneity of genomes in total RNA/DNA. Mycoplasma is also prevalent in clinical samples, and the capability to identify viral pathogen in mycoplasma or other microorganisms contained samples by NGS is necessary.

To sum up, we have fully characterized the presence of mycoplasma in our samples in the revised manuscript (Finding sections, highlighted in yellow, Additional files 2, 3 and 4), and we suggest that the contamination of mycoplasma would not affect the genome recovery of the viral genome.

## Additional files

Additional file 1: Figure S1. Schematic diagram of experimental design and analysis. A549, human alveolar adenocarcinoma cell line. Pretreatments of background depletion (BD) and/or whole-transcriptome amplification (WTA) were applied to mixed samples before library preparation. (DOCX 147 kb) Additional file 2: Flu and Mycoplasma reference genome datasets. (XLSX 8840 kb) Additional file 3: Figure S2. The distribution of total reads alignments. The total reads of three samples with a 0.55 % expected proportion of H1N1 within mixed RNA samples were first aligned to reference genomes of human (UCSC hg19) by using Bowtie2 and BLASTn with default parameters. The unaligned reads were then aligned to a dataset including reference genomes of Mycoplasma (313 sequences, NCBI genome database), bacterial (3022 sequences, NCBI genome database), flu (246,715 sequences, EpiFlu), other viral (1,757,357 sequences, NCBI genome database), and the whole NCBI nucleotide (nt) database by using Bowtie2 with default parameters. The Mycoplasma reads ratios for three samples were No pretreatment: 1.39 %, BD: 1.80 %, BD + WTA(8 h): 0.14 %. In the category of ‘Other’, we found that most of assignment could be considered as artificial alignments (chicken, rabbit, fruit fly, vector, etc.) which might be attributed to sequence homology. Undefined: failed to be aligned. (DOCX 130 kb) Additional file 4: Table S1. Ratios of mycoplasma-aligned reads in samples. (DOCX 16 kb) Additional file 5: Figure S3. Serotyping by HA and NA segments. Expected proportions of H1N1 within mixed RNA samples of were 0.55 % (a) and 1.5 % (b). (DOCX 124 kb) Additional file 6: Table S2. Primers used for RT-PCR amplification of H1N1 genomic segments (DOCX 22 kb) Additional file 7: Table S3. Single Nucleotide variations detected by next-generation sequencing and Sanger sequencing (DOCX 20 kb) Additional file 8: Table S4. The coefficient of variation for whole genome and each segment. (DOCX 18 kb) Additional file 9: Table S5. The missed and over-amplified nucleotide motifs with enrichment analysis. (DOCX 179 kb)

## Abbreviations

BD: Background depletion
WTA: Whole transcriptome amplification
CV: Coefficient of variation
