# Supplementary material for: Direct next-generation sequencing of virus-human mixed samples without pretreatment is favorable to recover virus genome
Source: Biol Direct. 2016 Jan 12;11:3. doi: 10.1186/s13062-016-0105-x (PMC4710016; doi:10.1186/s13062-016-0105-x)
Supplement: Additional file 1: Figure S1. — Schematic diagram of experimental design and analysis. A549, human alveolar adenocarcinoma cell line. Pretreatments of background depletion (BD) and/or whole-transcriptome amplification (WTA) were applied to mixed samples before library preparation. (DOCX 147 kb) [file 13062_2016_105_MOESM1_ESM.docx]

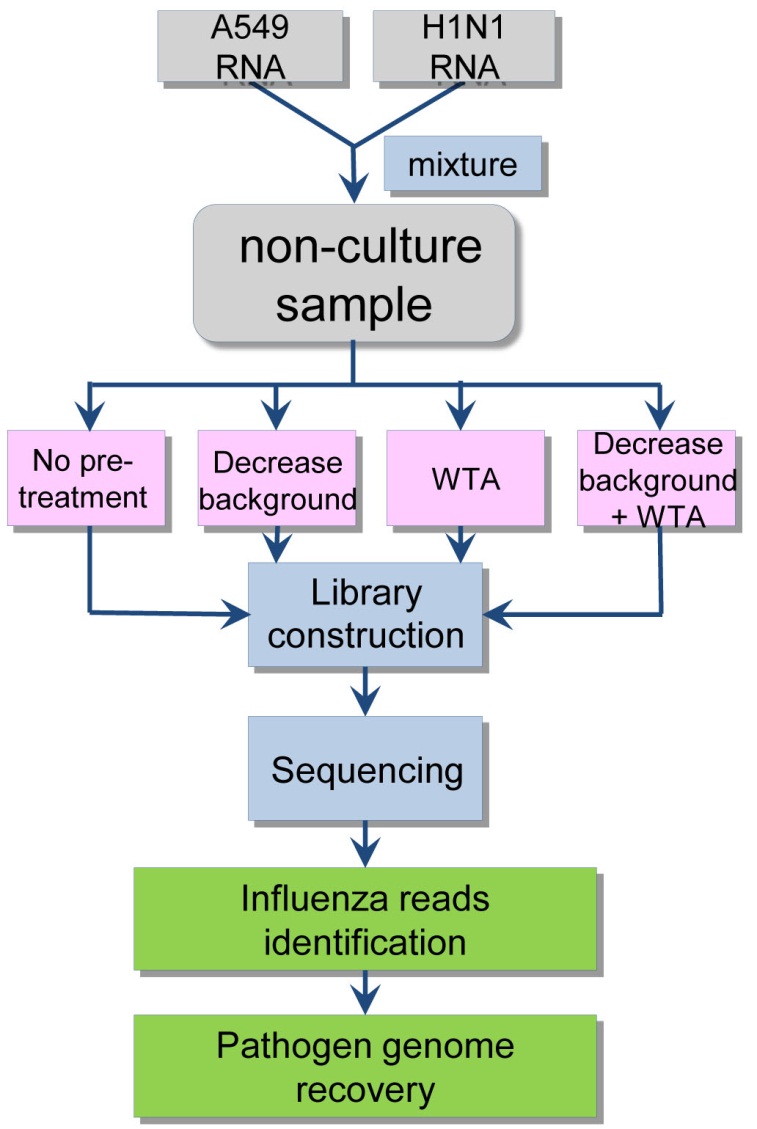


**Figure S1 Schematic diagram of experimental design and analysis.** A549, human alveolar adenocarcinoma cell line. Pretreatments of background depletion (BD) and/or whole-transcriptome amplification (WTA) were applied to mixed samples before library preparation.
