# Supplementary material for: Direct next-generation sequencing of virus-human mixed samples without pretreatment is favorable to recover virus genome
Source: Biol Direct. 2016 Jan 12;11:3. doi: 10.1186/s13062-016-0105-x (PMC4710016; doi:10.1186/s13062-016-0105-x)
Supplement: Additional file 3: Figure S2. — The distribution of total reads alignments. The total reads of three samples with a 0.55 % expected proportion of H1N1 within mixed RNA samples were first aligned to reference genomes of human (UCSC hg19) by using Bowtie2 and BLASTn with default parameters. The unaligned reads were then aligned to a dataset including reference genomes of Mycoplasma (313 sequences, NCBI genome database), bacterial (3022 sequences, NCBI genome database), flu (246,715 sequences, EpiFlu), other viral (1,757,357 sequences, NCBI genome database), and the whole NCBI nucleotide (nt) database by using Bowtie2 with default parameters. The Mycoplasma reads ratios for three samples were No pretreatment: 1.39 %, BD: 1.80 %, BD + WTA(8 h): 0.14 %. In the category of ‘Other’, we found that most of assignment could be considered as artificial alignments (chicken, rabbit, fruit fly, vector, etc.) which might be attributed to sequence homology. Undefined: failed to be aligned. (DOCX 130 kb) [file 13062_2016_105_MOESM3_ESM.docx]

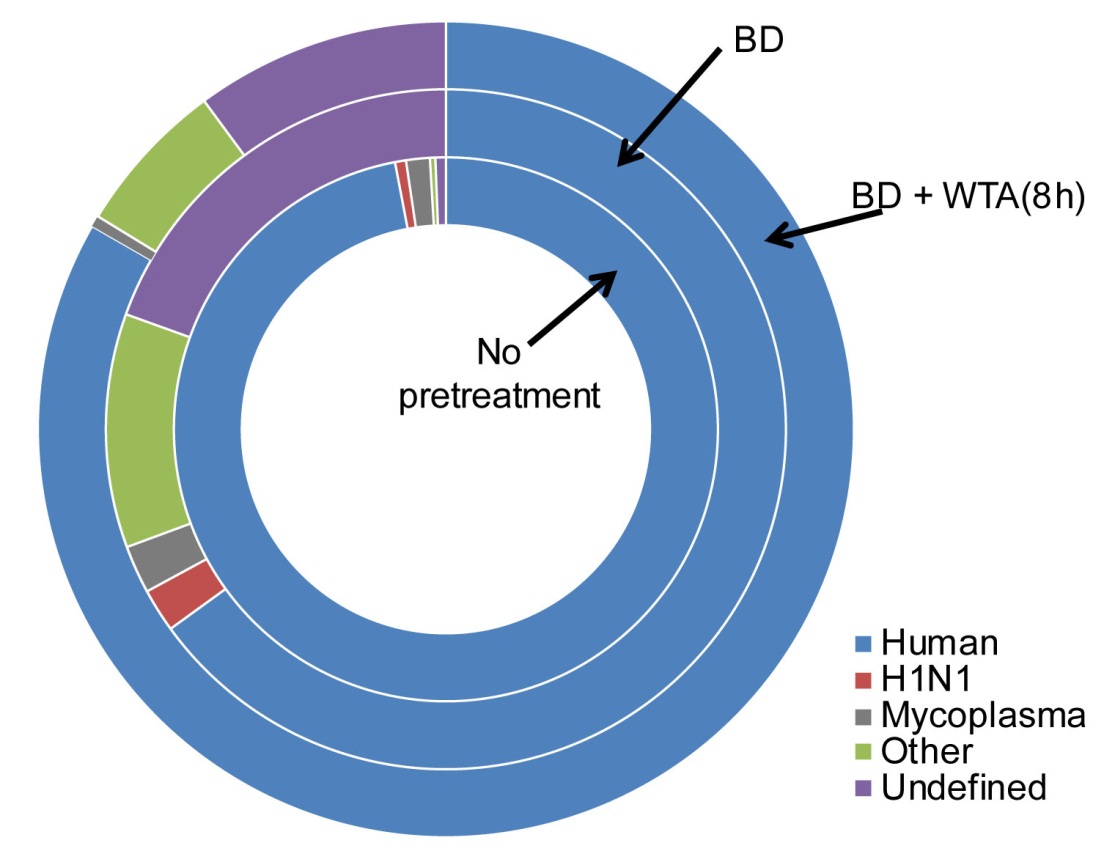


**Figure S2 The distribution of total reads alignments.** The total reads of three samples with a 0.55% expected proportion of H1N1 within mixed RNA samples were first aligned to reference genomes of human (UCSC hg19) by using Bowtie2 and BLASTn with default parameters. The unaligned reads were then aligned to a dataset including reference genomes of Mycoplasma (313 sequences, NCBI genome database), bacterial (3,022 sequences, NCBI genome database), flu (246,715 sequences, EpiFlu), other viral (1,757,357 sequences, NCBI genome database), and the whole NCBI nucleotide (nt) database by using Bowtie2 with default parameters. The Mycoplasma reads ratios for three samples were No pretreatment: 1.39%, BD: 1.80%, BD + WTA(8h): 0.14%. In the category of ‘Other’, we found that most of assignment could be considered as artificial alignments (chicken, rabbit, fruit fly, vector, etc.) which might be attributed to sequence homology. Undefined: failed to be aligned.
