# Supplementary material for: Direct next-generation sequencing of virus-human mixed samples without pretreatment is favorable to recover virus genome
Source: Biol Direct. 2016 Jan 12;11:3. doi: 10.1186/s13062-016-0105-x (PMC4710016; doi:10.1186/s13062-016-0105-x)
Supplement: Additional file 4: Table S1. — Ratios of mycoplasma-aligned reads in samples. (DOCX 16 kb) [file 13062_2016_105_MOESM4_ESM.docx]

**Table S1 Ratios of mycoplasma-aligned reads in samples.** After removal of the host-aligned reads, NGS reads were aligned to a dataset composed of 313 mycoplasma sequences (see Additional file 2) by using Bowtie2 (in the end-to-end, single-end mode, other parameters as default). The host-removed reads were also used as input for a metagenomics analysis by using both PathSeq and Kraken with default parameters. All reads assigned to mycoplasma (by bowtie2, PathSeq, and/or Kraken) were counted up to calculate the ratio of mycoplasma contamination for each sample.

| Treatment^a^ | Total reads | Mycoplasma reads ratio (%) |
| --- | --- | --- |
| BD (0.55%) | 2,373,019 | 1.80 |
| No pretreatment (0.55%) | 4,056,532 | 1.39 |
| BD + 8-h WTA (0.55%) | 4,606,715 | 0.40 |
| 8-h WTA (0.55%) | 5,270,948 | 0.14 |
| No pretreatment (1.50%) | 8,389,402 | 1.36 |
| BD + 2-h WTA (1.50%) | 10,251,680 | 5.05 |

^a^Expected proportions of H1N1 within mixed RNA samples are indicated in parentheses.
