# Supplementary material for: Direct next-generation sequencing of virus-human mixed samples without pretreatment is favorable to recover virus genome
Source: Biol Direct. 2016 Jan 12;11:3. doi: 10.1186/s13062-016-0105-x (PMC4710016; doi:10.1186/s13062-016-0105-x)
Supplement: Additional file 5: Figure S3. — Serotyping by HA and NA segments. Expected proportions of H1N1 within mixed RNA samples of were 0.55 % (a) and 1.5 % (b). (DOCX 124 kb) [file 13062_2016_105_MOESM5_ESM.docx]

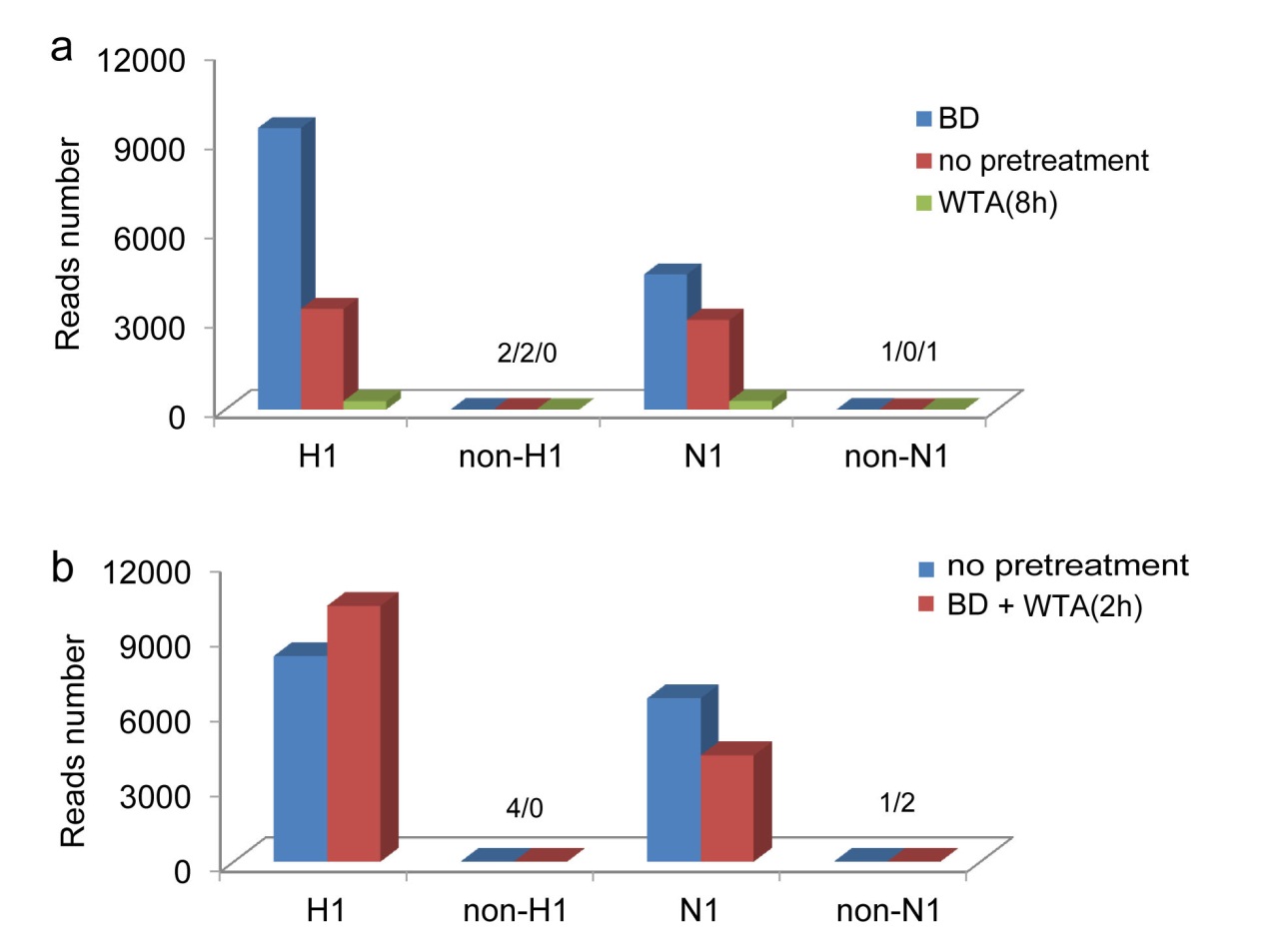


**Figure S3 Serotyping by HA and NA segments.** Expected proportions of H1N1 within mixed RNA samples were 0.55% (a) and 1.5% (b).
