# Supplementary material for: Direct next-generation sequencing of virus-human mixed samples without pretreatment is favorable to recover virus genome
Source: Biol Direct. 2016 Jan 12;11:3. doi: 10.1186/s13062-016-0105-x (PMC4710016; doi:10.1186/s13062-016-0105-x)
Supplement: Additional file 6: Table S2. — Primers used for RT-PCR amplification of H1N1 genomic segments (DOCX 22 kb) [file 13062_2016_105_MOESM6_ESM.docx]

**Table S2 Primers used for RT-PCR amplification of H1N1 genomic segments**

| Segemnt | Primers | Sequence (5'to3') | Primer length | Nucleotide position | Product size (bp) |
| --- | --- | --- | --- | --- | --- |
| NS | NS-1-F | AGCAAAAGCAGGGTGACAAAGA | 22 | 1-22 | 713 |
|  | NS-1-R | TTCTGTCCCAATTGCTCTCGCC | 22 | 713-692 |  |
|  | NS-2-F | GACATGACCCTCGAGGAAATG | 21 | 300-320 | 591 |
|  | NS-2-R | AGTAGAAACAAGGGTGTTT | 19 | 890-872 |  |
| MP | MP-1-F | AGCAAAAGCAGGTAGATATTTAA | 22 | 1-22 | 701 |
|  | MP-1-R | TGGAGCTAGGATGAGTCCCAAT | 22 | 701-680 |  |
|  | MP-2-F | CCGAACAACATGGATAGAGC | 20 | 293-312 | 735 |
|  | MP-2-R | AGTAGAAACAAGGTAGTTT | 19 | 1027-1009 |  |
| NA | NA-1-F | AGCAAAAGCAGGAGTTTAAAAT | 22 | 1-22 | 751 |
|  | NA-1-R | TCGGTCATTACAGTAAAGCAAG | 22 | 751-730 |  |
|  | NA-2-F | AATGTGCATGTGTAAATGGTTC | 22 | 709-730 | 750 |
|  | NA-2-R | AGTAGAAACAAGGAGTTT | 18 | 1458-1441 |  |
| NP | NP-1-F | AGCAAAAGCAGGGTAGATAATC | 22 | 1-22 | 800 |
|  | NP-1-R | TCAGCGTTCCCTGGGTTTCGAC | 22 | 800-779 |  |
|  | NP-2-F | GGCAATGATGGATCAAGTAAG | 21 | 753-773 | 813 |
|  | NP-2-R | AGTAGAAACAAGGGTATTT | 19 | 1565-1547 |  |
| HA | HA-1-F | AGCAAAAGCAGGGGAAAACAAA | 22 | 1-22 | 750 |
|  | HA-1-R | GATCCCTCACTTTGGGTCTTAT | 22 | 750-729 |  |
|  | HA-2-F | ATTCATACCCAAAGCTCAGCA | 21 | 550-570 | 736 |
|  | HA-2-R | TCCAGGTGGTTGAACTCTTTA | 21 | 1285-1265 |  |
|  | HA-3-F | CAGGATTGAGGAATGTCCCGTC | 22 | 1030-1051 | 748 |
|  | HA-3-R | AGTAGAAACAAGGGTGTTT | 19 | 1777-1769 |  |
| PA | PA-1-F | AGCAAAAGCAGGTACTGATCCA | 22 | 1-22 | 750 |
|  | PA-1-R | AATGCAGCCGTTCGGCTCGAAT | 22 | 750-729 |  |
|  | PA-2-F | GTAGATGGATTCGAGCCGAACG | 22 | 721-742 | 766 |
|  | PA-2-R | TGCTTATCATTGGGATCAGCTG | 22 | 1486-1465 |  |
|  | PA-3-F | GCCATGGATGACTTTCAGCTG | 21 | 1450-1470 | 784 |
|  | PA-3-R | AGTAGAAACAAGGTACTTT | 19 | 2233-2215 |  |
| PB1 | PB1-1-F | AGCAAAAGCAGGCAAACCATTT | 22 | 1-22 | 783 |
|  | PB1-1-R | GTATACGAAACCTCTAATCTGC | 22 | 783-762 |  |
|  | PB1-2-F | GGATGCAGATTAGAGGTTTCGT | 22 | 758-779 | 801 |
|  | PB1-2-R | AGCTGGGTAGCTCCATGCTAA | 21 | 1558-1538 |  |
|  | PB1-3-F | GCATGGAGCTACCCAGCTTTGG | 22 | 1541-1562 | 801 |
|  | PB1-3-R | AGTAGAAACAAGGCATTTT | 18 | 2341-2324 |  |
| PB2 | PB2-1-F | AGCAAAAGCAGGTCAAATATAT | 22 | 1-22 | 796 |
|  | PB2-1-R | GGTCAACATCATCATTTCTCAC | 22 | 796-775 |  |
|  | PB2-2-F | GTGAGAAATGATGATGTTGACC | 22 | 775-796 | 767 |
|  | PB2-2-R | GTACGTTCCCTCTTTGATCTC | 21 | 1541-1562 |  |
|  | PB2-3-F | GAGATCAAAGAGGGAACGTAC | 21 | 1541-1561 | 801 |
|  | PB2-3-R | AGTAGAAACAAGGTCGTTT | 19 | 2341-2323 |  |
