# Supplementary material for: Direct next-generation sequencing of virus-human mixed samples without pretreatment is favorable to recover virus genome
Source: Biol Direct. 2016 Jan 12;11:3. doi: 10.1186/s13062-016-0105-x (PMC4710016; doi:10.1186/s13062-016-0105-x)
Supplement: Additional file 7: Table S3. — Single Nucleotide variations detected by next-generation sequencing and Sanger sequencing (DOCX 20 kb) [file 13062_2016_105_MOESM7_ESM.docx]

**Table S3 Single Nucleotide variations detected by next-generation sequencing and Sanger sequencing**

| Segment | Position | Allele in Reference^a^ | Variation |
| --- | --- | --- | --- |
| NA | 1177 | A | G |
| NP | 2 | G | T |
| NP | 957 | C | T |
| HA | 464 | C | T |
| HA | 549 | A | G |
| HA | 748 | A | G |
| PB1 | 46 | C | A |
| PB1 | 228 | G | A |

^a^Reference: strain A/Changchun/01/2009(H1N1) (accession No. JN032403 - JN032410, NCBI Nucleotide database).
