# Supplementary material for: Direct next-generation sequencing of virus-human mixed samples without pretreatment is favorable to recover virus genome
Source: Biol Direct. 2016 Jan 12;11:3. doi: 10.1186/s13062-016-0105-x (PMC4710016; doi:10.1186/s13062-016-0105-x)
Supplement: Additional file 8: Table S4. — The coefficient of variation for whole genome and each segment. (DOCX 18 kb) [file 13062_2016_105_MOESM8_ESM.docx]

**Table S4 The coefficient of variation of site sequencing depth for whole genome and each segment**

| Treatment^a^ | Segment CV | | | | | | | | Genome  CV | p value^b^ |
| --- | --- | --- | --- | --- | --- | --- | --- | --- | --- | --- |
|  | HA | MP | NA | NP | NS | PA | PB1 | PB2 |  |  |
| No retreatment (0.55%) | 0.33 | 0.49 | 0.54 | 0.48 | 0.79 | 0.39 | 0.44 | 0.35 | 0.49 | — |
| BD (0.55%) | 0.60 | 0.82 | 0.92 | 1.06 | 1.20 | 0.69 | 0.92 | 0.58 | 1.03 | 0.008 |
| 8-h WTA (0.55%) | 0.63 | 0.60 | 0.80 | 1.06 | 1.55 | 0.69 | 0.54 | 0.46 | 1.59 | 0.008 |
| No retreatment (1.50%) | 0.40 | 0.47 | 0.51 | 0.46 | 0.58 | 0.41 | 0.42 | 0.33 | 0.47 | — |
| BD + 2-h WTA (1.50%) | 0.93 | 0.39 | 1.08 | 1.18 | 0.63 | 0.43 | 0.46 | 0.45 | 1.05 | 0.028 |

^a^Expected proportions of H1N1 within mixed RNA samples are indicated in parentheses.

^b^The p value was calculated by Wilcoxon rank sum test with “No pretreatment”.
