# Supplementary material for: Direct next-generation sequencing of virus-human mixed samples without pretreatment is favorable to recover virus genome
Source: Biol Direct. 2016 Jan 12;11:3. doi: 10.1186/s13062-016-0105-x (PMC4710016; doi:10.1186/s13062-016-0105-x)
Supplement: Additional file 9: Table S5. — The missed and over-amplified nucleotide motifs with enrichment analysis. (DOCX 179 kb) [file 13062_2016_105_MOESM9_ESM.docx]

**Table S5 The missed and over-amplified nucleotide motifs with enrichment analysis**

| **Motifs identified from missed region** | | | | | |
| --- | --- | --- | --- | --- | --- |
| Motif | | Pretreatment^a^ | Occurrences^*^ in | | p value^d^ |
|  |  |  | missed region^b^ | genome  (13,632bp)^c^ |  |
| 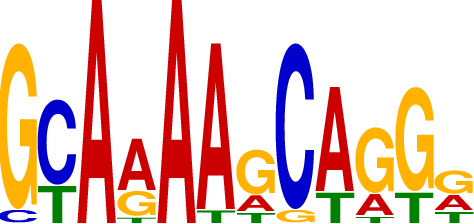 | | BD (0.55%)  8-h WTA (0.55%)  BD + 2-h WTA (1.50%) (1.50%) (1.50%) | 7 (3,235 bp)  6 (2,841 bp)  8 (3,031 bp) | 13 | 0.013  0.048  0.025 |
| 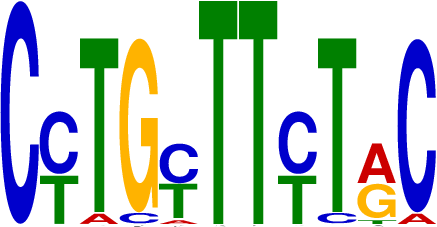 | | BD (0.55%)  8-h WTA (0.55%) | 6 (3,235 bp)  5 (2,841 bp) | 9 | 0.009  0.044 |
| 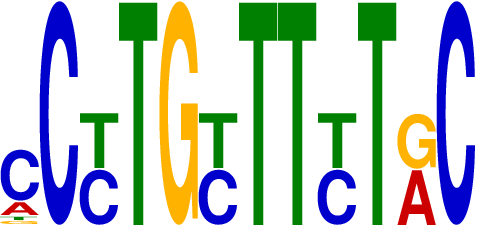 | | BD (0.55%)  8-h WTA (0.55%) | 6 (3,235 bp)  5 (2,841 bp) | 10 | 0.017  0.039 |
| 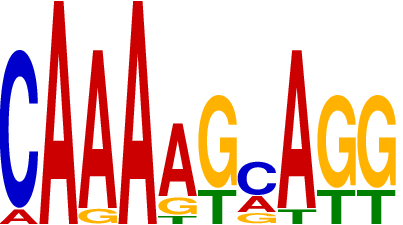 | | BD (0.55%)  8-h WTA (0.55%) | 12 (3,235 bp)  8 (2,841 bp) | 18 | 3.4e-5  0.020 |
| 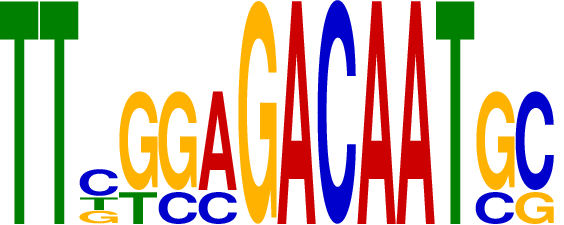 | | 8-h WTA (0.55%)  BD + 2-h WTA (1.50%) | 4 (2,841 bp)  4 (3,031 bp) | 5 | 0.008  0.008 |
| 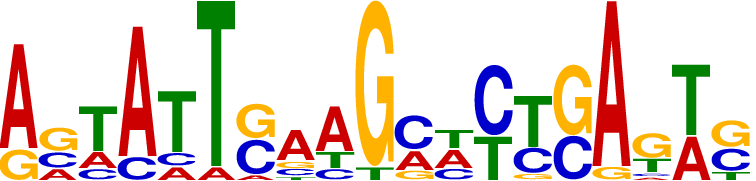 | | BD (0.55%) | 10 (3,235 bp) | 15 | 0.001 |
| 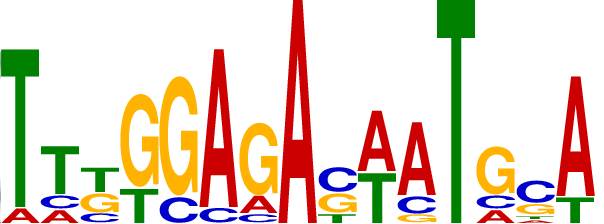 | | BD + 2-h WTA (1.50%) | 9 (3,031 bp) | 11 | 4.6e-5 |
| **Motifs identified from over-amplified region** | | | | | |
| Motif | Pretreatment^a^ | | Occurrences^*^ in | | p value^d^ |
|  |  |  | over-amplified region^b^ | # in genome  (13,632bp)^c^ |  |
| 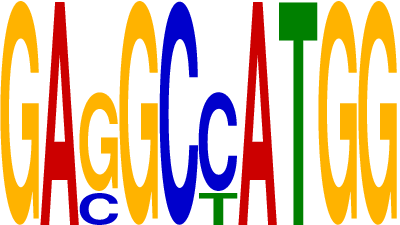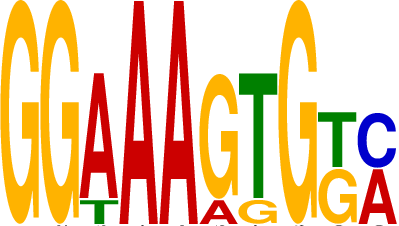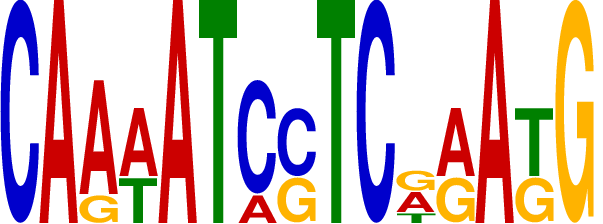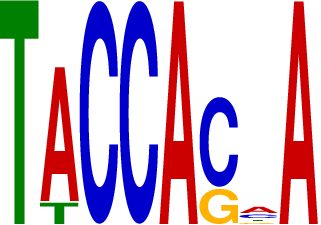 | BD (0.55%)  BD + 2-h WTA (1.50%) | | 6 (5,217 bp)  6 (3,976 bp) | 7 | 0.015  0.003 |
|  | BD (0.55%) | | 7 (5,217 bp) | 12 | 0.033 |
|  | BD (0.55%) | | 6 (5,217 bp) | 8 | 0.040 |
|  | 8-h WTA (0.55%) | | 6 (3,937 bp) | 9 | 0.021 |
| 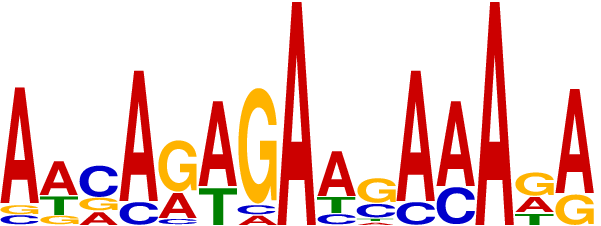 | 8-h WTA (0.55%) | | 13 (3,937 bp) | 19 | 4.0e-4 |

^*^Motifs whose occurrence time on the whole genome was fewer than 5 were excluded.

^a^Expected proportions of H1N1 within mixed RNA samples are indicated in parentheses.

^b^Length of the missed/over-amplified region determined by genome uniformity.

^c^Length of H1N1 reference genome.

^d^The p value was calculated by using Fisher’s exact test.
